# Intratumour Diversity of Chromosome Copy Numbers in Neuroblastoma Mediated by On-Going Chromosome Loss from a Polyploid State

**DOI:** 10.1371/journal.pone.0059268

**Published:** 2013-03-21

**Authors:** Gisela Lundberg, Yuesheng Jin, Daniel Sehic, Ingrid Øra, Rogier Versteeg, David Gisselsson

**Affiliations:** 1 Department of Clinical Genetics, Lund University, Skåne University and Regional Laboratories, Lund, Sweden; 2 Department of Paediatric Oncology and Haematology, Lund University, Skåne University Hospital, Lund, Sweden; 3 Department of Human Genetics, Academic Medical Center, University of Amsterdam, Amsterdam, The Netherlands; 4 Department of Pathology, Skåne University and Regional Laboratories, Lund, Sweden; Duke University, United States of America

## Abstract

Neuroblastomas **(**NBs) are tumours of the sympathetic nervous system accounting for 8–10% of paediatric cancers. NBs exhibit extensive intertumour genetic heterogeneity, but their extent of intratumour genetic diversity has remained unexplored. We aimed to assess intratumour genetic variation in NBs with a focus on whole chromosome changes and their underlying mechanism. Allelic ratios obtained by SNP-array data from 30 aneuploid primary NBs and NB cell lines were used to quantify the size of clones harbouring specific genomic imbalances. In 13 cases, this was supplemented by fluorescence *in situ* hybridisation to assess copy number diversity in detail. Computer simulations of different mitotic segregation errors, single cell cloning, analysis of mitotic figures, and time lapse imaging of dividing NB cells were used to infer the most likely mechanism behind intratumour variation in chromosome number. Combined SNP array and FISH analyses showed that all cases exhibited higher inter-cellular copy number variation than non-neoplastic control tissue, with up to 75% of tumour cells showing non-modal chromosome copy numbers. Comparisons of copy number profiles, resulting from simulations of different segregation errors to genomic profiles of 120 NBs indicated that loss of chromosomes from a tetraploid state was more likely than other mechanisms to explain numerical aberrations in NB. This was supported by a high frequency of lagging chromosomes at anaphase and polyploidisation events in growing NB cells. The dynamic nature of numerical aberrations was corroborated further by detecting substantial copy number diversity in cell populations grown from single NB cells. We conclude that aneuploid NBs typically show extensive intratumour chromosome copy number diversity, and that this phenomenon is most likely explained by continuous loss of chromosomes from a polyploid state.

## Introduction

One of the hallmarks of human solid tumours is genomic instability, arising from aberrations of the normal biological mechanisms that repair, replicate and transmit the genome [1]. Faithful segregation of chromosomes to daughter cells during mitosis maintains chromosome stability and a diploid genome. Disrupted control of this system may lead to chromosomal mis-segregation and an ensuing pattern of chromosomal instability (CIN) and intratumour diversity with respect to the copy numbers of individual chromosomes. CIN has also been less frequently used to describe the presence of structural aberrations [2], [3] and in inherited syndromes with increased risk of cancer [4]. However, the mechanisms that contribute to structural complexity, on the one hand, and numerical changes, on the other hand, are largely distinct. Structural rearrangements can be caused by abnormal DNA repair pathways that lead to errors in end-joining of double-stranded DNA. Structural rearrangements may also occur through telomere-mediated events, where abnormally short telomeres are recognized as DNA breaks leading to chromosomal alterations when DNA-repair pathways are activated [5]. In contrast, alterations in chromosome number (aneuploidy) typically result from abnormalities in mitotic spindle assembly checkpoint function [6], centrosome duplication [7], [8], and microtubule stability [9].

Aneuploidy is a very common feature in solid tumours [10]–[15]. A high degree of aneuploidy is typically associated with poor prognosis, particularly in adult cancers [16]. One well-known exception from this is the childhood tumour neuroblastoma (NB), where a near triploid karyotype is typically interconnected with a better clinical outcome [17]. NB is the most frequently occurring extra-cranial solid tumour in children and about 90% of children with the disease are diagnosed within the first 5 years of life. NBs exhibit extensive intertumour genetic heterogeneity, and are traditionally subdivided into three clinical-genetic subtypes, based on the pattern of somatic chromosome alterations [18]. Type 1 tumours are characterised by a hyperdiploid to near-triploid chromosome number with no/few structural aberrations, and absence of *MYCN* amplification. Type 2A tumours have near-diploid or near-tetraploid karyotypes dominated by structural rearrangements, most prominently 17q gain and 11q deletions, still with absence of *MYCN* amplification. In contrast, type 2B tumours are signified by amplification of *MYCN,* often in conjunction with 1p deletion and 17q gain in a near-diploid or near-tetraploid background. While type 1 NBs typically occur in children <18 months of age and have an excellent prognosis, type 2A and B tumours tend to occur in older children and are associated with a less favourable outcome. Accordingly, numerical chromosome aberrations/aneuploidy are most prevalent and most pronounced in type 1 NBs. However, less dramatic aneuploidy, often limited to a few trisomies and monosomies, can also be found in the other subtypes. Of the 273 NB cases reported in the Mitelman Database of Chromosome Aberrations and Gene Fusions in Cancer (http://cgap.nci.nih. gov/Chromosomes/Mitelman), 174 have a non-diploid or non-tetraploid karyotype, implying that more than 60% of NBs are aneuploid.

We have previously reported that telomere length abnormalities are frequently present in NB and have linked these to structural chromosome instability [19]. However, the mechanisms underlying aneuploidy in NB remain largely unexplored. Neither has it been thoroughly assessed whether individual cells or cellular subpopulations present in the same tumour vary in copy number in a fashion similar to adult tumours exhibiting CIN. Such intercellular variation may be of importance for tumour progression and chemotherapy resistance on the basis of clonal evolution, and could also have a role in explaining regional variation in biology and morphology within the same tumour. The aim of the present study was to perform a survey of the prevalence and extent of intratumour diversity in NBs with respect to numerical aberrations and to deduce the most likely route to aneuploidy in this tumour type. For this purpose we used a combination of experimental and bio-informatic techniques.

## Materials and Methods

### Ethics Statement

Written consent was given by each patient’s parents for documentation, biological studies and analysis of medical data. The study was approved by the Lund University Ethics Committee (L119/03, L289/11) and by the Review Board of the Academic Medical Center in Amsterdam.

### Tissue Samples and Cell Lines

Frozen tumour material was obtained from the biobanks of the Departments of Clinical Genetics and Human Genetics at Lund University (Lund, Sweden) and the Academic Medical Center (Amsterdam, The Netherlands), respectively. Only tumours classified as primary NBs were included, after having undergone histopathological review. All patients were treated according to the European protocol active at the time. The follow-up time ranged from 91 days (dead of the disease) up to 18 years. Established NB cell lines were obtained, from LGC Standards (Teddington, UK) and DSMZ (Braunschweig, Germany). All analysed NB cases were sub-divided into clinical-genetic subtypes according to Brodeur [18] as follows. Type 1: non-*MYCN* amplified hyperdiploid to near triploid tumour dominated by whole chromosome changes. Type 2A: non-*MYCN* amplified near-diploid or near-tetraploid tumour dominated by structural chromosome changes. Type 2B: *MYCN* amplified near-diploid or near-tetraploid tumour. Cell line culture, fixation and chromosome preparation for fluorescence in situ hybridization (FISH) were performed according to standard methods previously described [20].

### Scoring of Mitotic Segregation Errors

Cell lines were cultured on chamber slides and washed in phosphate buffered saline (PBS). The slides were fixed at −20°C in methanol and dried at room temperature. Cell nuclei and chromosomes were counter-stained with DAPI. Using a fluorescence microscope, anaphase bridges, lagging chromosomes and multipolar mitoses were scored as previously described [21].

### FISH

For the purpose of assessing variation in whole chromosome copy number, centromere-specific probes for chromosomes 1, 2, 4, 7, 11, 12, 16, 17 and 18 were obtained (Abbott Laboratories, Abbott Park, IL). Single copy probes for 1p, 11q and 17q (Abbott Laboratories) were also obtained from the same source. FISH was performed according to standard procedures as previously described [20]. The number of signals in each cell was manually scored in digital images acquired by a CCD camera coupled to an epi-fluorescence microscope. A normal fibroblast sample (NIGMS/Corriell Cell Repositories, Camden, NJ) served as a technical control for the overall hybridization efficiency for each centromere probe used and this was found to be close to 100%. For each specimen, including the normal fibroblasts, at least 200 nuclei were analyzed.

### Single-cell Cloning

A suspension of 10 ml containing 100 cells was prepared from each of three cell lines. The suspension was diluted in steps and transferred into a 96 well plate, incubated for growth in standard medium. The following day, each well was examined using an inverted light microscope. Wells containing only one cell were marked with a permanent marker and monitored every second day until approximately 1000 cells were present. The cells were then trypsinized and placed in chamber slides for approximately one week, then harvested according to methods previously described [20]. At least 200 nuclei in three clones from each cell line were analysed by FISH.

### Time Lapse Imaging

Time-lapse imaging was performed using a Nikon Eclipse Ti camera (Nikon Instruments Europe B.V. Laan van Kronenburg 2, 1183AS Amstelveen, The Netherlands). The total time between frames was 5 minutes.

### Single Nucleotide Polymorphism (SNP) Array Analysis

For high-resolution detection of genomic imbalances in cell lines and frozen tumour tissue, 300 ng of DNA extracted using standard methods (DNeasy Blood & Tissue Kit, Qiagen, Valencia, CA) was then hybridized to Illumina HumanCNV660/Omnia BeadChips (Illumina Inc., San Diego, CA) according to the manufacturer’s specifications. Allele specific fluorescent signals were first normalized using a proprietary algorithm in the Illumina BeadStudio software (Illumina Inc). Normalized allelic intensity values were thereafter exported and subjected to an additional normalization step using the tQN-software [22]. The tQN software also estimates B-allele frequencies (BAF) for each SNP based on a set of reference genotype clusters. For identification of imbalances, the BAF segmentation software was used, in which BAF-values are transformed into mirrored BAF (mBAF) values followed by removal of non-informative homozygous SNPs [23]. Segments with mBAF values >0.55 were classified as being in allelic imbalance. Segments with log2 ratio >0.073 were classified as genomic gains, those with log2 ratios <0.080 as genomic losses, and those with log2 ratios between these boundaries as copy number neutral genomic imbalances. Segments were fused if the interspersed genomic distance was <1 Mb and the difference in mBAF values between the segments was <0.1. Constitutional copy number variants were excluded from the final data by comparison to the Database of Genomic Variants (http://projects.tcag.ca/variation/; last access Aug. 1 2011). Bioinformatic interpretation of array profiles was supplemented by manual analysis in cases of high complexity or ambiguity. In cases of ambiguous ploidy, karyotyping data was used as a reference.

mBAF values were also used to estimate the proportion of sampled DNA containing the respective genomic imbalances, calculated according to Staaf et al. [23]. This approach has previously been shown to accurately predict the proportion of tumour DNA carrying genomic imbalances in samples with known ploidy levels [23], [24]. The chosen mBAF threshold of 0.56 for calling allelic imbalances allowed detection of hemizygous losses (*e.g*. monosomies) present in clones exceeding 20% of and single copy gains (*e.g.* trisomies) in clones exceeding 25% of sampled DNA. The minimum and maximum mBAF values within each abnormal segment were used build confidence intervals for the proportion of sample DNA containing the abnormality in question. Clone size spans were calculated as the difference in relative DNA content between the most and least prevalent genomic aberrations. Amplified sequences (>5 copies) could not be quantified by the above approach and were omitted from the analysis. Complete raw data from the BAF segmentation algorithm, as well as curated segments corresponding to genomic imbalances with their estimated relative tumour DNA content are deposited as Dataset S1. Raw genotype data are not provided in order to protect patient privacy.

### Touch Preparations

Frozen tumour material was immediately placed on dry ice and a 2–3 mm piece carved from each biopsy using a scalpel. The piece was then picked up using sterilized tweezers and gently pressed against a microscopy slide. The amount of imprinted cells was validated via phase-contrast microscopy. Prior to FISH, the slides were placed in fixative, one part glacial acetic acid and three parts methanol, for ten minutes and then dried in room temperature. The slides were then kept in the freezer at −20°C. As a normal control, post-mortem anonymized adrenal tissue from the biobank at the Department of Pathology in Lund, was used – having undergone the same procedure of freezing and thawing as NB tissue.

### In Silico Simulations

The stochastic models used to simulate mitotic chromosome segregation errors have been previously described [16]. In brief, distributions of whole chromosome copy numbers (*i.e.* the relative frequencies of monosomies, disomies, trisomies, tetrasomies, pentasomies and higher polysomies) in NBs were tested against four previously described main types of aberrant mitosis occurring in tumours, including (1) loss of chromosomes from the tetraploid level, (2) sequential sister chromatid non-disjunction, (3) tripolar mitosis coupled to sister chromatid non-disjunction, and (4) tripolar mitosis coupled to incomplete cytokinesis. Whole chromosome copy number profiles from NBs containing numerical chromosome aberrations were obtained from two independent datasets. The first was the Mitelman Database of Chromosome Aberrations and Gene Fusions in Cancer (http://cgap.nci.nih.gov/Chromosomes/Mitelman; Dec. 18 2011) providing abnormal karyotypes from 273 NBs. Excluding karyotypes from recurrent tumours, from adults (>18 years of age), karyotypes with no numerical changes, incomplete karyotypes (inc), and karyotypes with markers (mar) or ring chromosomes (r), there remained 52 cases with high-quality karyotypes from paediatric primary NBs (Dataset S2). For clinical-genetic classification, the presence of double minutes (dmin) or hsr (homogeneously staining regions) in the karyotype was assumed to be equivalent to *MYCN* amplification. The second dataset was based on SNP array (Illumina HumanCNV660 or Omni BeadChip) analysis of >100 NBs accessible through the R2 Microarray Analysis and Visualization Platform (http://hgserver1.amc.nl/cgi-bin/r2/main.cgi?&species=hs; Dec. 14 2011) from which the 68 cases containing whole chromosome copy number aberration were extracted (Dataset S3).

To compare the whole chromosome copy number profiles expected from each type of abnormal mitosis to empirical data, a set of R-based algorithms (R.app GUI 1.33 5582 Leopard build 32-bit, R Foundation for Statistical Computing, 2009) were constructed where chromosomes were assumed to segregate normally or abnormally, independently of each other in a fashion according to the type of aberrant mitosis being simulated [16]. Each individual NB case was compared to each of the four models by simulating monoclonal expansion from 10,000 virtual pre-neoplastic cells with either a normal diploid (sequential sister-chromatid non-disjunction; the two types of tripolar mitosis) or a normal tetraploid karyotype (loss from tetraploidy; the two types of tripolar mitosis). Each of these original 10,000 cells with a balanced chromosome complement was then set to evolve step-wise according to its specific type of chromosome segregation error until its chromosome number was the same as of the case being evaluated. Cells that obtained nullisomies (0 copies of a chromosome) were eliminated by replacement with random sampling from the remaining population. The resulting dataset from 10,000 virtual tumours with the same chromosome number was used to calculate the expected prevalence of the specific copy number distribution (i.*e.* the distribution of monosomies, disomies, trisomies etc.) found in the real tumour being evaluated. Performing such simulations for all tumours present in a specific clinical-genetic subtype also generated an overall expected relative distribution of chromosome copy numbers according to each of the abnormal mitotic processes tested. This parameter was used together with the expected prevalence values of observed copy number distributions to assess which type of mitotic aberration best predicted the chromosome aberrations found in each clinical-genetic subtype.

### Statistical Analysis of Empirical Data

Averages of normal-like distributions were compared using two-tailed Student’s t-test. Fisher’s exact test, two-tailed, was used for nonparametric tests (STATISTICA version 20.0.0, IBM, 2011, CA).

## Results

### Evidence of Chromosomal Instability in NB Cell Lines

**Figure 1:**
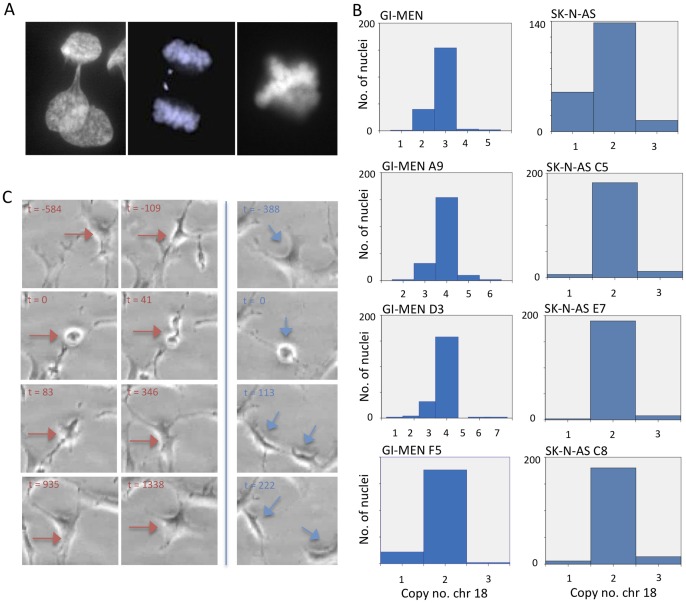
Ongoing chromosomal instability in NB cell lines. *(A)* Visualisation of three aberration types during mitosis in GI-MEN, ana-telophase bridging (left), chromatid lagging (centre) and a multipolar mitosis (right). *(B)* Copy number distributions of chromosome 18 in GI-MEN and SK-N-AS original cultures and three single cell-based clones derived from them. In the GI-MEN original culture (top left), the modal number was three, whereas in the clones, two exhibit a modal copy number of four (A9 and D3) and the third (F5) a modal copy number of two. In SK-N-AS a modal number of two is maintained in the clones (C5, E7 and C8). (*C*) Time lapse phase contrast microscopy of dividing GI-MEN cells showing a cell (left panel; red arrow) entering mitosis (t = 0 min) and following through until anaphase (t = 41 min), but then failing to undergo cytokinesis (followed for 24 h post mitosis). Adjacent cells underwent normal cell divisions (right panel; blue arrow). Time is annotated in minutes from maximum mitotic round-up. Total time for video monitoring was 48 h 35 min. See Video S1 for full time lapse session.

To experimentally explore to what extent mitotic segregation errors that could contribute to aneuploidy were present in NB cells, we first analysed mitotic figures in five established NB cell lines. All lines showed aberrations during mitosis. Chromosome loss through lagging was the most common type of aberration with a mean of 8% of anaphase cells, compared to 5% for bridges and 1% for multipolar mitosis, indicating that chromosome loss is a common and ongoing process in growing NB cells (Table 1; Figure 1A). In contrast, control fibroblasts subjected to the same type of analysis showed cell division anomalies in only 1% of mitoses, all of which consisted of anaphase bridges (data not shown).

**Table 1:** Mitotic segregation errors^1^.

	AB/TB	Lagging	Multipolar m.
**GI-ME-N**	7	17	3
**SK-N-AS**	1	8	0
**IMR 32**	3	7	0
**SK-N-SH**	0	2	0
**SK-N-FI**	12	4	3

1 Showing the different mitotic errors in percentage of anaphase/telophase figures for anaphase/telophase bridges (AB/TB) and lagging, and of all mitotic figures for multipolar mitoses.

To explore whether chromosomal instability was an ongoing phenomenon that could generate tumour cell sub-populations with different chromosome numbers, we then created single cell clones from one *MYCN* amplified NB cell line (GI-MEN) and one non-amplified cell line with multiple segmental aberrations (SK-N-AS), and from hTERT transduced fibroblasts as a stable karyotype control. To compare numerical variation within single cell clones, FISH with centromere probes for chromosome 11, 17 and 18 was performed (Table 2; Figure 1B). As expected, the single cell clones showed less copy number diversity than their respective original cell populations. Overall, GI-MEN clones showed a trend towards higher chromosome copy number diversity than SK-N-AS clones (mean 14% compared to 8% of cells with copy number outside the modal value; p = 0.05), consistent with the higher rate of mitotic segregation errors in the former cell line. All chromosomes and segments assessed in GI-MEN and SK-N-AS showed a significantly higher degree of copy number heterogeneity compared to the single cell clones made from fibroblasts. This supported that genomic variation was an ongoing phenomenon in NB cells, with respect to both structural and numerical changes.

**Table 2:** Single cell clones.

	CEP 11	CEP 17	CEP 18	1p	17q
**Fibroblasts control**					
**n = **	2	2	2	2	2
**non-modal (%)**	0.5	0	0	0	0
**GI-MEN mother line**					
**n = **	2	2	3	2	2
**non-modal (%)**	**51**	**36**	**23**	**8**	**48**
**GI-MEN F5**					
**n = **	5	2	2	2	5
**non-modal (%)**	**8**	**5**	**12**	**5**	**34**
**GI-MEN A9**					
**n = **	6	2	4	2	6
**non-modal (%)**	**30**	**9**	**23**	**18**	**43**
**GI-MEN D3**					
**n = **	5	2	4	2	5
**non-modal (%)**	**5**	**12**	**21**	**1**	**42**
**SK-NAS mother line**					
**n = **	2	1	3	2	4
**non-modal (%)**	**14**	**26**	**32**	**16**	**73**
**SK-N-AS C8**					
**n = **	2	1	2	2	4
**non-modal (%)**	**11**	**6**	**13**	**10**	**66**
**SK-N-AS E7**					
**n = **	2	1	2	2	4
**non-modal (%)**	**5**	**6**	**8**	**12**	**50**
**SK-N-AS C5**					
**n = **	2	1	2	2	4
**non-modal (%)**	**5**	**6**	**8**	**12**	**50**
**Mean** **non-modal (%)**	11	7	14	10	48

1 The first row shows the modal number and the second the percentage of nuclei having a different copy number than that of the modal number. Results above the mean non-modal fraction for all probes in fibroblasts +3 standard deviations (0.77%) are denoted in bold type, corresponding to a significant prevalence of cells with non-modal chromosome number.

It has been well established that whole genome duplication/tetraploidisation, is a distinct aspect of genomic tumours instability that may contribute to clonal evolution [25]. Two of the main routes towards tetraploidisation are fusion of two diploid cells and mitosis with cytokinetic failure, respectively, both leading to cells with a duplicated chromosome complement [25]. We performed phase contrast time-lapse microscopy of growing GI-MEN cells to assess the presence of either of these routes. In total 52 cell divisions were followed from prophase through telophase within a total time frame of 6 days of time lapse imaging of >100 cells. No event of cell fusion was observed. However, 6% of mitotic GI-MEN cells failed to undergo cytokinesis (Figure 1C and Video S1). When a similar number of mitotic hTERT transduced fibroblasts (60 cell divisions) was followed, no cytokinetic failure was found (data not shown). Thus, polyploidisation through failed cell division can occur regularly in NB cells, in parallel to evolution by mis-segregation of individual chromosomes.

### Intratumor Genetic Diversity in NB Tumours and Cell Lines

**Figure 2:**
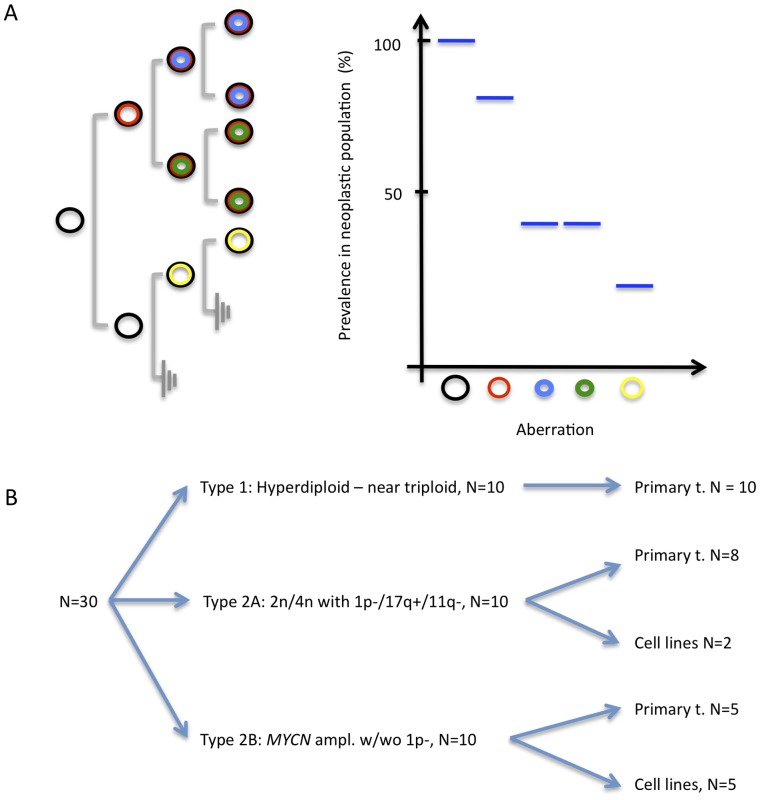
Basic principles and material for survey of diversity by SNP array analysis. *(A)* A monoclonal cellular proliferation with ongoing genomic instability (left panel) will result in a genetically heterogeneous population, where specific genetic aberrations (denoted by circles) will be present at different prevalence values. By SNP array analysis, these values can be approximated from mBAF data for genomic aberrations that lead to allelic imbalances (right panel; details in Materials and Methods). The detected prevalence levels will to some extent reflect the temporal development of genetic aberrations because early aberrations (black and red circles) will tend to have higher prevalence values than later genetic abnormalities. However, the relationship between prevalence and time of occurrence cannot be strictly relied upon, as it will be confounded by variable growth rates for different sub-clones (exemplified by blue, green, and yellow circles). *(B)* Cases selected for survey of genomic diversity by SNP-array, subdivided according to clinical-genetic subtype.

To make a broader survey to what extent intratumour genomic diversity was present in primary NB and NB cell lines, we then used allelic ratios (mBAF values) obtained by SNP array analyses to approximate the prevalence of tumour cells harbouring each detected allelic imbalance (Figure 2A). This approach will well approximate the proportion of tumour DNA carrying specific genomic imbalances down to approximately 20%, against either a normal cell background or a background of tumour cells with diverse sub-clonal genomic changes [23], [24]. From a larger cohort of approximately 100 NB tumours and cell lines analysed by SNP-array, we consecutively selected the 10 cases of each clinical-genetic subtype having the highest quality SNP array profile as quantified by baseline variation at the diploid level (Figure 2B).

**Figure 3:**
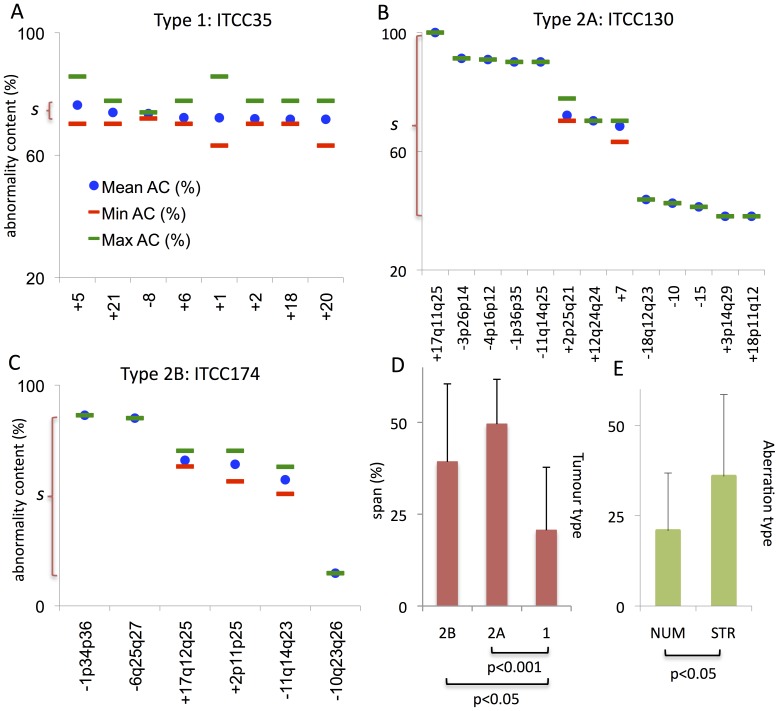
Intratumour genome diversity by SNP array analysis. *(A–C)* Representative examples of relative DNA content estimates from mBAF data for allelic imbalances in three NBs. The prevalence (DNA content) of each allelic imbalance is given as abnormality content (AC) plotted on the y-axis, while the type of aberration is specified along the x-axis. Red and green lines indicate maximum and minimum estimated DNA content. Segments affected by aberrations are specified according to cytogenetic nomenclature;+and - indicate gain and loss of a single copy, respectively. ITCC35 is a Type 1 NB exhibiting trisomy for seven chromosomes and monosomy for one, all of which are present at similar prevalence. ITCC130 is a type 2A tumour with multiple structural and numerical changes present at different prevalence levels, including 1p deletion, 11q deletion and 17q duplication. ITCC174 is a type 2B *MYCN* amplified NB with structural aberrations confined to three distinct prevalence levels. The prevalence span (*s*) of each case is denoted on the y axis. *(D)* Mean prevalence spans according to clinical-genetic type and *(E)* aberration type (NUM, numerical/whole chromosome changes; STR, structural changes); errors bars denote standard deviations.

Among these 30 cases, the detected aberrations in primary tumours varied in their relative tumour DNA content from approximately 100% down to 20% (Dataset S1). All cases showed at least one aberration represented in >50% of tumour DNA (exemplified in Figure 3A–C). As expected, the cell lines showed a trend towards a higher prevalence of abnormalities in tumour DNA, but differences were not significant, and DNA contents of aberrations ranged from 97% down to 27% in the cell lines. Because it can be assumed that all primary tumour samples were contaminated to some degree by non-neoplastic cells, the DNA content estimates cannot be used for valid assessment of intratumour genetic diversity. We therefore chose to quantify diversity as the DNA content span between the most and least frequent genomic imbalance in each case (Figure 3A–C). By this approach, all analysed cases exhibited some degree of intratumour clonal variation (Table 3), ranging from 5% to 72%. In comparison, non-neoplastic control tissue showed baseline variation corresponding to a span <1% extrapolated from mBAF values (data not shown). Including all types of aberrations, the DNA content span was narrower (p<0.05) for Type 1 tumours (mean 21%) compared to tumours belonging to Types 2A (50%) and 2B (39%; Figure 3D). The difference was even more pronounced when only primary NB tumours were compared (means 20% in type 1 vs. 60% and 58% in 2A and B; p<0.001).

**Table 3:** Clinical data and genome diversity assessed by spans of clone prevalence (DNA content).

Case^1^	Age(days)	Sex(M/F)^2^	Stage^3^	Time torelapse(days)	Follow-uptime(days)	Out-come^4^	Location	Ploidy	Totalno. ofsegments	Numerialchanges	Structuralchanges	Prevalencespan (%)
**Type 1: Whole chromosome changes**												
ITCC35	51	F	4S	–	4686	NED	abdomen	2n–3n	8	8	0	5
NRC1	340	M	3	–	1495	NED	paravertebral–abdominal	3n–5n	15	15	0	10
NRC10	107	M	1	–	5659	NED	adrenal gland	3n–4n	13	13	0	10
NRC3	194	F	2	–	2280	NED	paravertebral-thoracal	3n	7	7	0	13
ITCC44	0	F	2A	–	3195	NED	adrenal gland	2n–3n	12	11	2	15
ITCC111	80	F	4S	–	3897	NED	adrenal gland	2n–3n	11	11	0	16
NRC5	5	M	2B	66	2443	NED	paravertebral-thoracal	2n–3n	10	10	0	20
ITCC9	385	F	2A	160	5871	NED	paravertebral-thoracal	2n–3n	11	11	0	23
ITCC2	41	M	2A	–	4845	NED	paravertebral-thoracal	3n	12	10	2	30
ITCC38	183	M	4S	–	4945	NED	adrenal gland	2n–3n	14	12	2	65
**Type 2A: Structural changes, no ** ***MYCN*** ** amplification**												
SK-N-SH	–	–	–	–	–	–	–	2n	5	1	4	26
ITCC130	1339	M	4	515	515	DOD	paravertebral-thoracal	2n	11	0	11	42
ITCC10	281	M	4	–	6450	NED	adrenal gland	2n	6	0	6	45
NRC13	7	M	2	–	2475	NED	adrenal gland	4n	9	6	3	46
NRC14	1815	M	4	494	1573	NED	cervical	4n	9	0	9	48
ITCC36	1364	M	4	91	136	DOD	adrenal gland	2n	10	1	9	50
NRC7	2408	F	3	191	1779	DOD	abdomen	2n	14	10	4	50
ITCC29	304	M	1	676	2884	AWD	paravertebral-thoracal	2n	5	0	5	56
ITCC13	1009	F	4	450	480	DOD	adrenal gland	2n	13	2	10	62
SK-NA-S	–	–	–	–	–	–	–	2n	19	3	16	71
**Type 2B: M** ***YCN*** ** amplification**												
**NRC8**	721	F	4	–	11	DOC	abdomen	4n	6	4	2	7
**NRC4**	441	M	4	1182	2389	NED	adrenal gland	2n	4	0	4	19
**IMR32**	–	–	–	–	–	–	–	2n	10	1	9	23
**SKNBE**	–	–	–	–	–	–	–	4n	26	6	20	29
**NRC11**	697	F	4	340	1360	DOD	adrenal gland	2n	4	0	4	35
**ITCC31**	531	M	4	599	853	DOD	adrenal gland	2n	6	2	4	38
**LAN1**	–	–	–	–	–	–	–	4n	25	7	18	46
**GI-M-EN**	–	–	–	–	–	–	–	4n	22	4	18	61
**SK-N-FI**	–	–	–	–	–	–	–	2n	17	1	16	64
**ITCC174**	818	M	4	329	400	DOD	adrenal gland	2n	6	0	6	72

1 NRC, collected from Lund University Hospital, Sweden; ITCC, collected from Academic Medical Center, Amsterdam, the Netherlands.

2 F, female; M, male.

3 According to the International Neuroblastoma Staging System.

4 Status at latest follow-up annotated as: NED, no evidence of disease; DOD, dead of disease; DOC, dead of complications; AWD, alive with disease.

We then compared the span of whole chromosome copy number changes within each case with that of structural aberrations (Figure 3E). There was a significantly narrower span for numerical aberrations in general (mean 21%) than for structural aberrations (mean 36%; p<0.05 irrespective of inclusion of cell lines), indicating that the narrower span in Type 1 tumours compared to Types 2A and 2B was due to the manifold higher prevalence of whole chromosome changes in the former type as compared to the high prevalence of structural changes in the latter types. Indeed, the total DNA content span correlated positively to the number of structural aberrations (Pearson r = 0.55) and negatively to the number of numerical aberrations (r = −0.48). Furthermore, an average of 78% of the detected numerical aberrations were within the same DNA content level (estimated from minimum and maximum mBAF values; Dataset S1) as the majority of aberrations present in the same case, most likely corresponding to the majority clone. In contrast, only 52% of the structural changes were confined to the majority clone (p<0.01 with either inclusion or exclusion of cell lines).

In summary, SNP array-based estimates of intratumour diversity revealed a difference between tumours dominated by whole chromosome changes (Type 1) and those dominated by structural aberrations (Types 2A and B), with a clear hierarchy of clones corresponding to aberrations with different DNA content being more common in the latter two.

### Intercellular Genetic Diversity in NB Tumours and Cell Lines

**Figure 4:**
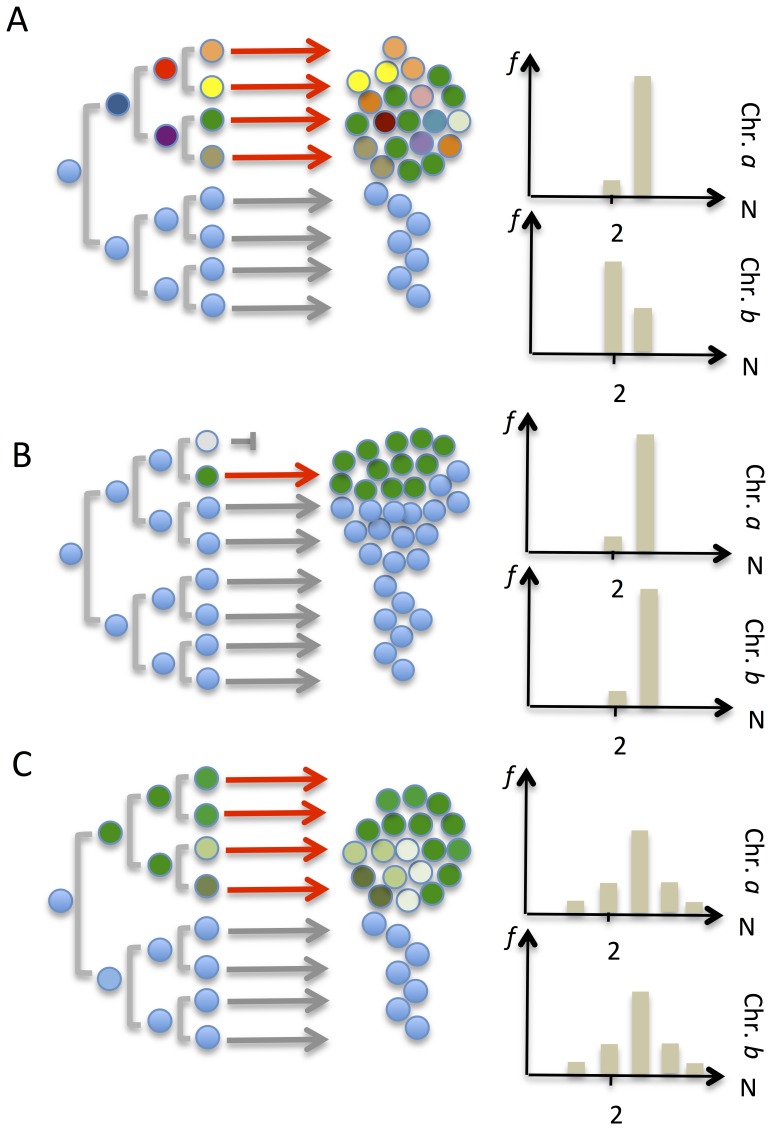
Models of genome diversity. *(A)* Ongoing genomic instability with evolution of distinct sub-clones during tumour development. Circles of different colours represent cells with partly different genotypes; light blue circles are cells with a normal karyotype. There will be a high probability for variation in prevalence for genomic changes that occur at different steps during clonal evolution. In the right panel, this is exemplified by trisomies for chromosomes *a* and *b*, where trisomy *a* occurs early in the process and will be present at a high prevalence, while *b* occurs later with presence only in some cells. The relative frequencies (*f*) of copy numbers are denoted on the y axis and the segment copy number on the x axis. *(B)* A single event (such as an unbalanced mitosis or a genetic bottleneck) leading to clonal expansion from a single cell, with no ensuing genetic instability. If trisomies for chromosomes *a* and *b* are present in the cell of origin, their prevalence values will be similar in the resulting cell population, while cells not descendants from the cell of origin should have normal copy number. *(C)* A single event followed by genetic instability of a stochastic nature, resulting in near-random variation around a modal value for chromosomes *A* and *B*, both being trisomic in the cell of origin. Prevalence estimates from SNP array analysis will be able to distinguish *A* from *B* and *C*, but not *B* from *C*.

**Figure 5:**
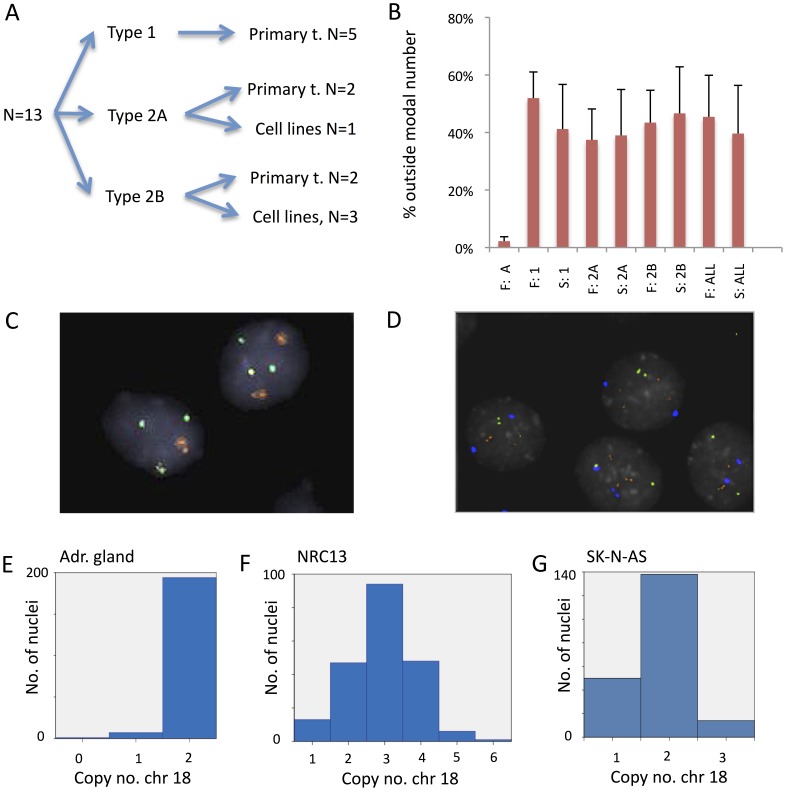
Intratumor genome diversity by FISH analysis. *(A)* Cases selected for survey of genomic diversity by FISH analysis, subdivided according to clinical-genetic subtype. *(B)* Mean proportion of cells with a non-modal copy number for whole chromosomes, taking all FISH-analysed chromosomes into account, with data sub-divided according to clinical-genetic subtypes (1, 2A, 2B). Data from FISH (F) and SNP array analyses are shown side-by-side. FISH data from normal post-mortem adrenal tissue (A) is included as a control. Error bars denote standard deviations. (*C*) FISH analysis of tumour touch preparation (NRC7), here showing centromere probe 1 in orange (2 copies in both cells) and centromere 12 in green (3 copies). (*D*) FISH analysis of the GI-MEN cell line, here displaying chromosome 18 centromere in blue, 1p in green and 17q in orange. (*E and F*) The distribution of chromosome 18 copy numbers by FISH in the adrenal gland control and NRC 13.

Prevalence span estimation from SNP array data is expected to reveal clear clonal population strata within a tumour biopsy or cell line, if such are present. However, it cannot discern clones with a DNA content <20% and thus gives little information on cell-to-cell variation. Furthermore, it cannot clearly differentiate contamination of diploid cells from a background of cells with stochastic variation in copy number centred around the modal chromosome numbers of a major clone (Figure 4). Hence, while the SNP array analysis found little evidence for significant genome diversity in Type 1 NBs it did not strictly exclude stochastic or near stochastic variation at the cellular level. In order to assess intercellular copy number in detail, we therefore selected a subset of the tumours analysed by SNP array for further investigation by interphase FISH on tumour touch preparations (Figure 5A). To be able to assess the intratumour diversity of whole chromosome numbers we used centromere probes for chromosome 1, 2, 4, 7, 11, 12, 16, 17 and 18. For each case, FISH experiments were tailored to cover chromosomes/genome segments with aberrations showing both high and low clonal prevalence by SNP array analysis. For comparison, we also used probes for 1p, 11q and 17q, being segments commonly affected by structural aberrations in NB.

Compared to normal control adrenal tissue, all cell lines and all but one of the tumours (NRC4) showed elevated intercellular copy-number variation for the majority of the analysed chromosomes/segments, with up to 75% and 73% of tumour cells showing non-modal copy numbers in primary tumours and cell lines, respectively (Table S1). There was no difference between the fraction of cells with non-modal chromosome numbers when whole chromosomes and chromosome segments were compared (mean 36% and 34%, respectively). The fraction of cells with non-modal chromosome copy numbers found by FISH was highly similar to estimations by SNP array (mBAF) data (Figure 5B). Consistent with the SNP array data, FISH analysis showed a trend for whole chromosomes to have similar fractions of cells with non-modal copy numbers within the same case, while structural changes showed a more dispersed pattern (mean 17% and 34% respectively; p = 0.02). However, in the majority of NBs (12/13), all whole chromosome copy numbers evaluated by centromeric probes showed significantly higher fractions of cells with non-modal copy numbers than did normal adrenal tissue (Figure 5F; Table S1). This was inconsistent with the scenario expected if all numerical aberrations were confined to a single genetically homogeneous clone (Figure 4B). Instead, there was typically a presence of multiple small sub-populations with non-modal chromosome numbers, as predicted from near-stochastic variation around the modal value (Figure 4C). Thus, intratumour diversity appeared to be present for both numerical and structural chromosome aberrations in NB, but with a seemingly stochastic variation around a modal value for the former in contrast to the clear clonal hierarchies observed for the latter types of aberrations.

### Chromosome Loss from a Tetraploid Intermediate Best Explains the Pattern of Numerical Changes

**Figure 6:**
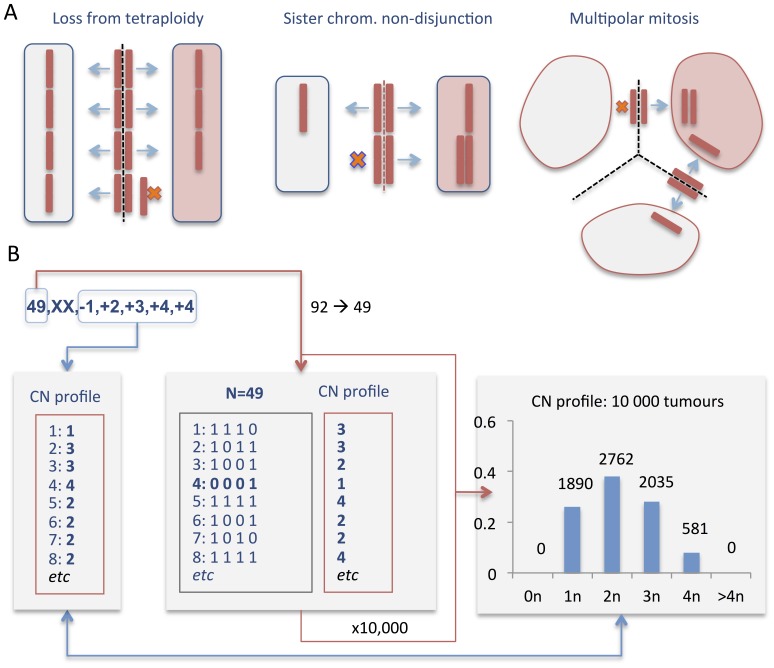
Models of mitotic instability. *(A)* Schematic overview of the types of mitotic segregation error evaluated for comparison against cytogenetic and SNP array data. Segregation errors were modelled by translating the most common outcome for each type of error into an algorithm: (1) Loss from tetraploidy was modelled as a process that sequentially and randomly creates loss of single chromatids from the tetraploid level [7]. (2) Sister chromatid non-disjunction was set up as a serial process creating one monosomic and one trisomic daughter cell in each step [24]. (3) Multipolar mitosis was modelled as generating three daughter cells with failure of proper sister chromatid separation, leading to a randomised distribution of chromatids to each daughter cell [34] as shown in the figure, or amphitelic segregation and cytokinetic failure leading to one daughter cell with chromosome gains and another with losses (not shown). *(B)* Example of modelling loss of tetraploidy for a tumour with a stem line karyotype containing 49 chromosomes with monosomy 1, trisomies 2 and 3, and tetrasomy 4. The chromosome number N = 49 is used as the target value for simulations of 10,000 tumour genomes formed by random loss of chromosomes from a chromosome number of 92 (tetraploid). Each of these virtual tumours will have a certain copy number (CN) profile (from 0–4), depending on the distribution of losses over its different chromosomes. The profiles of all 10,000 tumour stem lines are used to generate an overall CN profile for tumours with 49 chromosomes created by loss from tetraploidy. This dataset is then used to assess the expected prevalence of the specific CN profile of the original tumour, on the condition that it was created by loss from tetraploidy (lower blue arrow). To compare observed (cytogenetics/SNP array data) to expected (simulated) data for specific groups of NB cases, overall observed vs. overall expected CN profiles are also compared by Chi-square test (Figures 7 and 8).

The distinct sub-clonal stratification of structural changes found in NB by SNP array analysis is a common finding in cancer [26]. In several tumours, including NB, abnormal mitotic chromosome segregation such as anaphase bridging caused by telomere dysfunction has been suggested to be a major underlying mechanism of such intercellular variation in chromosome structure [27]. However, the mechanism causing variation in chromosome number has been little investigated in NB. To connect our findings of a near-stochastic variation in chromosome copy number in NB with possible mitotic errors, we used a statistical approach (Figure 6). By this method, the specific pattern of chromosome copy numbers (number of monosomies, disomies, trisomies, tetrasomies *etc.*) found in a cohort of tumour cases by cytogenetics or genomic arrays is compared to patterns expected from different types of mitotic error previously reported in tumour cells [16]. These mitotic errors include (1) chromosome loss from the tetraploid level, (2) sequential sister chromatid non-disjunction, and (3) multipolar mitosis. For evaluation of each reported tumour genome, 10,000 virtual diploid or tetraploid tumour stem lines are created and allowed to evolve according to each different model of mitotic segregation error, until a chromosome number identical to that of the reported tumour is reached. Details of simulations are provided in the legend to Figure 6 and in Materials and Methods.

For comparison of genomic patterns in NB with those expected from the respective types of chromosome segregation errors, we extracted high-quality genomic data from one cytogenetic and one SNP array-based dataset, resulting in 52 and 68 cases of NB with whole chromosome aberrations, respectively. The prevalence of different chromosome copy number profiles in these NBs were compared to those expected from the various types of chromosome segregation. In the first set of simulations, no restrictions on cell survival were imposed by chromosome number, except for elimination of cells with nullisomies. Under such conditions, none of the tested scenarios were able to replicate the overall distribution of copy number profiles in NB. More than 90% of observed copy number distributions showed an expected prevalence close to 0 (range 0–0.0001) and the overall observed copy number distributions poorly matched those expected from the simulations (p<0.0001; Chi Square test).

**Figure 7:**
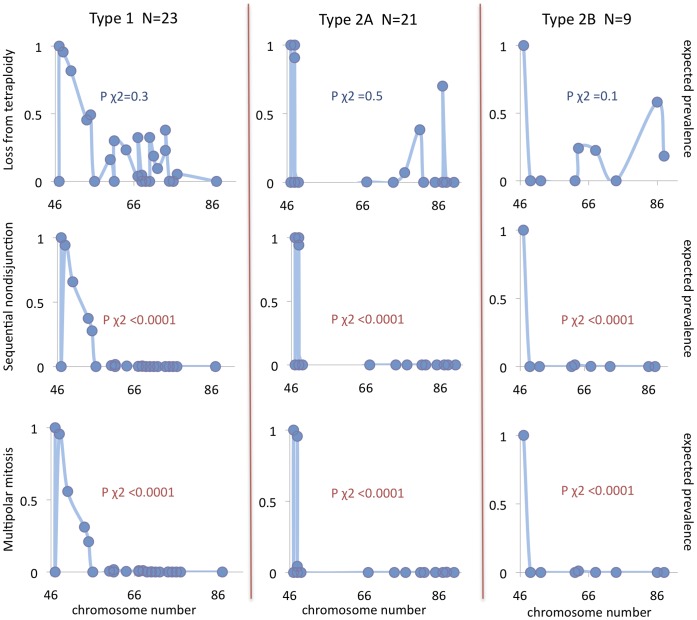
Expected frequencies of observed karyotypes for different types of mitotic instability. NB cases retrieved from the Mitelman Database of Chromosome Aberrations and Gene Fusions in Cancer were subdivided according to clinical-genetic subtype (vertical panels) and according to simulations of three different types of mitotic segregation error (horizontally). Each blue circle denotes a case, charted according to its chromosome number (x axis) and the expected prevalence of its CN profile (y axis) according to the type of segregation error being simulated. P values reflect the results of Chi square testing of the overall observed distribution in each subtype (cytogenetics) against the expected distribution (simulations). Monosomic and nullisomic cells were eliminated from all simulations. For multipolar mitosis, only results based on amphitelic segregation and cytokinetic failure are shown because modelling of randomised segregation resulted in prevalence values <1% for all CN profiles.

**Figure 8:**
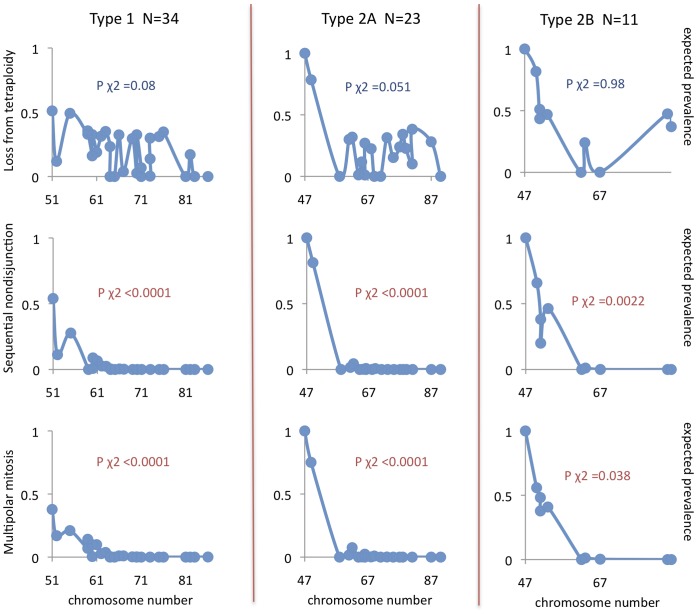
Expected frequencies of observed SNP array profiles for different types of mitotic instability. NB cases analyzed by SNP array were subdivided according to clinical-genetic subtype, analyzed, and annotated as described in Figure 7. Monosomic and nullisomic cells were eliminated from all simulations. For multipolar mitosis, only results based on amphitelic segregation and cytokinetic failure are shown because modelling of randomised segregation resulted in prevalence values <1% for all CN profiles.

In a second round of simulations, we took into account the fact that monosomies are very rare in NB genomes, being present in only around 12% of cases in our dataset, with no case showing >1 monosomy. Algorithms were then adjusted to impose a negative impact on cell survival from monosomy, replacing virtual tumours with at least one monosomy with a randomly sampled tumour from the entire set of 10,000 tumours. Renewed simulations showed that most types of mitotic segregation error still poorly predicted the observed copy number distributions (Figures 7 and 8). The type of mitotic error least compatible with the observed copy number distributions in NB was multipolar mitosis from a diploid state (mean expected prevalence ≈0 for both cytogenetic and SNP array data, with a distinctly different expected distribution at p<0.0001 by Chi Square test for both datasets), followed by multipolar mitosis from a tetraploid state (mean expected prevalence 14% and 19% for cytogenetic and SNP array data, respectively; p<0.0001 for both), sequential sister chromatid separation from a diploid state (16% and 18%; p<0.0001 for both), and sequential sister chromatid separation from a tetraploid state (16% and 17%; p<0.0001 for both). In contrast, loss of chromosomes from the tetraploid level produced copy number distributions with higher predicted prevalence values (27% and 33%) and an overall distribution of copy numbers profiles that was not significantly different from the observed ones (p>0.05 for both datasets). There were no differences between the clinical-genetic subtypes of NB, all of which showed copy number profiles best predicted by chromosome loss from the tetraploid state (Figures 7 and 8). However, for all tested models of mitotic segregation errors, the simulations showed highly similar expected copy number profiles for cases with only a few whole chromosome changes. Our findings that loss from tetraploidy best predicts the scenario of numerical changes in NB was therefore valid only for cases with >50 chromosomes.

### Modelling NB Genome Evolution as a Dynamic Process

**Figure 9:**
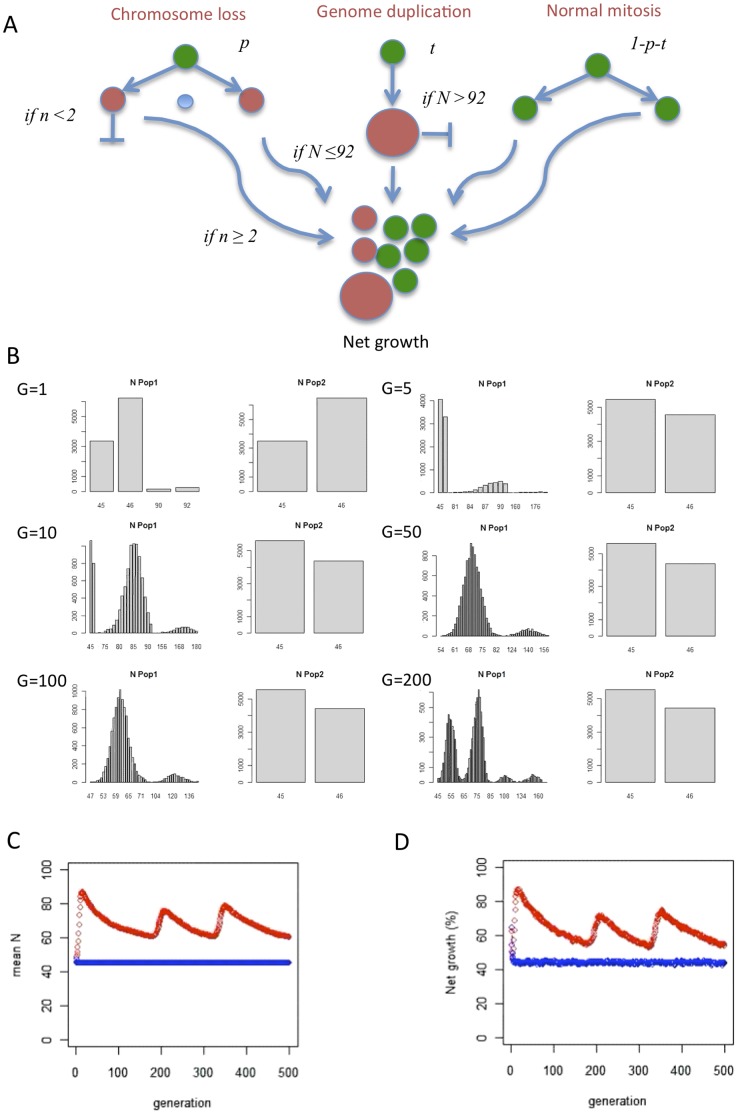
Dynamic modelling of NB genome evolution. *(A)* A virtual population of 10,000 cells with a normal diploid genome was set to evolve for 500 mitotic generations, with a probability *p* of losing a chromosome at each mitoses, the probability *t* to undergo whole genome duplication (typically tetraploidisation) through mitotic failure, and the probability *1-p-t* to undergo an error-free mitosis. The input values of *p* and *t* were obtained from experimental studies of GI-MEN. Cells with ≤1 copies of any chromosome (*n*) or a chromosome number (*N*) >92 were excluded from future generations. Net growth was estimated as the fraction of cells at each time point having the possibility of undergoing mitosis, i.e. cells without nullisomies, monosomies or high polyploidy (N>92). (*B*) Chromosome number evolution over the first 200 mitotic generations (G) for the entire population where the bars (y axis) denote the number of cells (N) having a certain chromosome number (x axis). For each generation, results are shown for a test population (Pop1; left panel) under conditions described in *A* and a control population (Pop2; right panel) where whole genome duplication cannot take place. Already at G = 10, Pop1 becomes dominated hypotetraploid cells, as cells with chromosome losses down to the monosomic level are outcompeted by those having undergone whole genome duplication. The dominance is then shifted to near-triploid cells (G = 50, G = 100). At G = 200 a hyperdiploid population co-exists with a hypotetraploid one as cells with chromosome losses down to the monosomic level are again outcompeted by others having undergone whole genome duplication. The full simulation is presented in Video S2. (*C*) Mean chromosome number (mean N) for the entire Pop1 (red line) over 500 generations show a fluctuation around the triploid level, while the control population (Pop2, blue line) is stably near-diploid. (*D*) Estimated net growth rate for Pop1 (red line) fluctuates in parallel to mean chromosome number and overall exceeds that of Pop2 (blue line), the latter accumulating a high fraction of cells with monosomies which are not expected to re-enter mitosis.

The finding that loss from tetraploidy was the model best explaining patterns in aneuploidy in NB was consistent with our initial *in vitro* data, showing that that both chromosome lagging and whole genome duplication were common events in NB cell lines. The finding of significant intercellular copy number diversity in NB clones derived from single cells further indicated that chromosomal instability was an ongoing process. Taken together, our findings suggested a rudimentary dynamic model for aneuploidy in NB, comprising whole-genome duplication, continuous chromosome loss, and selection against cells with monosomies. To test whether such a model would create the typically hyperdiploid-hypotetraploid karyotypes observed in highly aneuploid NBs, we simulated growth from a diploid pre-tumour population with a certain probability to undergo chromosome loss or whole genome duplication/polyploidisation at each mitosis (Figure 9A). Based on extended FISH-based cell division analysis of GI-MEN, the probability of chromosome loss was set to 4×10^−2^ per chromosome per mitosis (combined lagging and loss from non-disjunction; data not shown) and the frequency of polyploidization to around 6%. We imposed negative selection on cells having obtained nullisomies or monosomies, by complete elimination from further generations. Based on available cytogenetic data (Mitelman Database of Chromosome Aberrations and Gene Fusions in Cancer; http://cgap.nci.nih.gov/Chromosomes/Mitelman) we also imposed a similar drastic negative selection on highly polyploid cells (>92 chromosomes) as majority clones with such chromosome numbers occur only in approximately 2.5% of NBs (7 of 273 cases).

When virtual populations of 10,000 diploid pre-tumour cells were allowed to evolve over 500 mitotic generations using these conditions, identical results were produced by 10 independent simulation experiments: virtual cells losing chromosomes from a diploid state were rapidly out-competed by the descendants of other virtual cells that underwent tetraploidisation at an early stage (Figure 9B, G = 1–10; Video S2). This resulted in a growing population dominated by cells in the hypotetraploid range, gradually shifting through triploidy down towards hyperdiploidy (Figure 9B, G = 50–100). When reaching diploidy, a population shift, similar to that at early generations, occurred that again shifted the population to a hypotetraploid state. Most of the generation time for the population was spent at a peri-triploid state (Figure 9C), during which most of the extrapolated net tumour growth took place (Figure 9D). A control cell population not able to undergo whole genome duplication but otherwise grown under identical conditions, showed an accumulation of hypodiploid cells, existing in a dynamic equilibrium with diploid cells, reflecting a balance between the rate of chromosome loss and the survival advantage of cells without monosomies; no hyperdiploid cells formed in this population. Taken together, our dynamic model showed that continuous chromosome loss, combined with negative selection against cells with nullisomy and monosomy, and a capacity for whole genome duplication will consistently result in a dominance of tumour cells with chromosome numbers in the peri-triploid range – similar to the distribution found in most highly aneuploid NBs.

## Discussion

**Figure 10:**
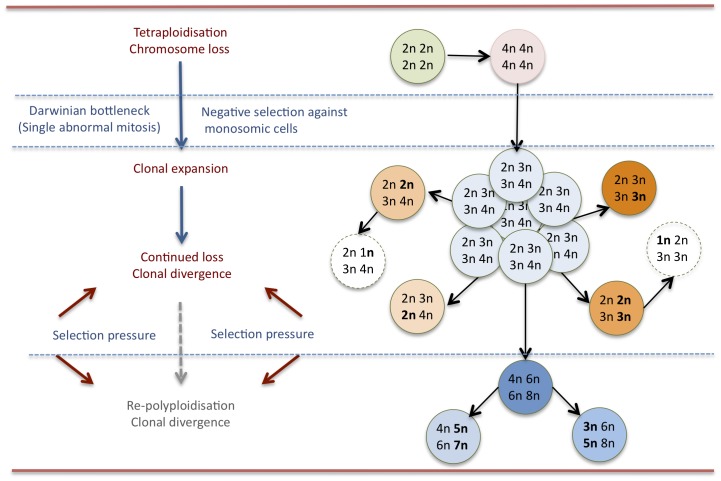
Suggested model for aneuploidy development in NB. A diploid pre-tumour cell undergoes tetraploidisation followed by/or in combination with chromosome loss until a copy number profile allowing clonal expansion is obtained; the present data do not fully exclude that this first step occurs through a single event even though later copy number changes appear to occur in a step-wise fashion. Chromosomes are continuously lost during clonal expansion leading to a population with copy number diversity, the composition of which is further modified by endogenous selection against cell with monosomies and nullisomies, as well as exogenous unknown factors. Further subclonal development unto a higher ploidy level may also occur through repeated whole genome duplications.

In the present study we show that intratumour diversity of large-scale genomic imbalances is a feature of most NBs, irrespective of clinical-genetic subtype. These results were arrived at by two independent methods, i.e. SNP array and FISH analysis. Although diversity was observed for both structural and numerical chromosome aberrations, the former were distributed in clonal hierarchies while the latter type of aberrations showed near-stochastic variation around modal numbers. Simulations indicated that this stochastic distribution most likely reflected a process of chromosome loss from a tetraploid state, which was supported by experimental data showing frequent chromosome loss and polyploidisation in NB cells. Taken together, our data converged into a complex dynamic model for aneuploidy development in NB, most relevant to tumours with chromosome numbers in the hyperdiploid-hypotetraploid range (Figure 10). To our knowledge, this is the first report of intratumour chromosome number variation and its underlying mechanisms in NB.

Our *in silico* approach for estimating the most likely mechanism behind numerical aberrations in NB yielded conclusive results but never the less has several inherent limitations. For example, it assumes that only one route of mitotic error is present in each tumour, which is likely to be an oversimplification [21]. Modelling a sequence of different types of segregation error is certainly feasible. On the other hand, this approach would provide a very large number of possible combinations even when only limited series of events is modelled. The construction of such *in silico* experiments would therefore lend itself to a high degree of subjectivity, for example in deciding how many subsequent errors should be allowed in a simulated sequence. Based on such considerations, we used a highly reductionist approach in the present paper. The *in silico* modelling yielded informative results only after negative selection against virtual cells with monosomies were introduced, based on extrapolation from their scarceness in cytogenetic and SNP array data. Finally, none of the applied models took into account the variable degree of positive and negative selection for different types of numerical aberrations, which is most probably present in NB as their pattern is clearly non-random [28], . However, similar analyses have been performed previously and were shown to correlate well to experimental data on cell division errors in tumour cells [16]. A similar type of analysis has also been performed for acute childhood leukemia [30], using the frequencies of uniparental disomies to validate different scenarios behind hyperdiploidy. However, that approach does not allow an equally fine-tuned distinction between the different scenarios of mitotic segregation error and was therefore not applied in the present study. For example, randomised segregation through multipolar mitosis from a diploid state cannot be distinguished from loss of chromosomes from the tetraploid level. Even though we found our simulation data on chromosome segregation errors to be consistent with experimental findings of chromosome lagging and polyploidisation, it should be stressed that (1) the loss-from-tetraploidy model provides only the best fit out of a few available models and there may be other mechanisms behind aneuploidy hitherto unknown that fit the data better, (2) the models only showed significant differences for tumours with >50 chromosomes and that the mechanism behind the creation of moderately hyperdiploid karyotypes was not resolved by the present study, and (3) individual NBs, even if having >50 chromosomes, may still obtain aneuploidy by a different route, as our approach evaluated only which single scenario that best fit the overall genomic data.

Our *in silico* simulations yielded data comparable to reported NB genomes only when strong selection against monosomic cells were introduced in the algorithms. Consistently, there was a very low prevalence of monosomies in the genomic profiles reported by SNP array and cytogenetics, and FISH analysis did not show a monosomic modal number for any of the whole chromosomes assessed in cell lines or primary tumours in the present study, except for chromosome 17 in SK-N-AS. Nevertheless, the loss from tetraploidy model suggested here stipulates that monosomies are continuously produced in growing tumour cell populations, which is consistent with our FISH data showing monosomic sub-populations for most of the assessed chromosomes. The low number of monosomies in tumour stem lines might be explained by monosomic cells lacking the necessary genomic material for proliferative survival. If so, whole genome duplication through failed mitosis or other mechanisms may confer sufficient extra genomic material to allow growth even in a context in which chromosome copies are continuously lost through genomic aberrations. The necessity to have such genomic “buffering capacity” to balance an inherent chromosomal instability could potentially explain why aneuploid NBs typically have chromosome numbers in the hyperdiploid to triploid range. However, a cell population subjected to constant whole chromosome loss will sooner or later reach a point when a high rate of monosomies are generated, in turn leading to reduced growth capacity if monosomy is associated with reduced cellular survival. The reaching of such a critical point could, in theory, explain why the typically highly aneuploid Type 1 (infants with stage 1 or 4s) NBs show an excellent prognosis and may even spontaneously regress [31], [32]. However, tumours having numerical aberrations in combination with amplification of *MYCN* or other structural chromosome changes generally do not share this favourable course of disease [17]. Possibly, this is due to a lower overall rate of chromosome loss in these tumours. As long as there is no stable *in vitro* system for Type 1 NBs available for comparison of mitotic segregation errors to Types 2A and 2B, this hypothesis cannot be further tested.

The thought of intratumour diversity of chromosome numbers based on cell division anomalies in NB is not new. Kaneko and Knudsen [33] suggested a theoretical model for the development of near-triploid NBs based on tripolar division of a tetraploid cell, assuming asymmetrical 2-3-3 amphitelic distribution of the eight chromatids from each tetrasomic chromosome. Later studies have so far failed to show experimentally the significance of this mechanism in human tumour cells. Instead, it has been shown that tripolar mitoses may either circumvent the spindle assembly checkpoint, leading to a high frequency of non-disjunction events and near-random distribution of chromatids to daughter cells [34]. Alternatively, the spindle assembly checkpoint can be satisfied and chromosomes will segregate amphitelically to the daughter cells in a tripolar fashion, but chromosomes will never the less be asymmetrically distributed because of cytokinetic failure, leading to one daughter cell with chromosome gains and another with chromosome losses [24]. Taken together, these data suggest that the scenario proposed by Kaneko and Knudsen is not a typical outcome of tripolar cell division. The data of the present study also fail to support this hypothesis in its original form, as our experimental observations show that the generation of numerical aberrations appears to be an ongoing process rather than a stable state based on a single event. Furthermore, our mathematical simulations of copy number distributions resulting from tripolar mitoses were poorer predictors of the actual scenario in NB than those based on continuous loss from the tetraploid level. However, it should be noted that the Kaneko-Knudsen model is consistent with our findings in the sense that it suggests an early tetraploid cell from which chromosomes are lost to create aneuploid tumours.

Chromosomes can be lost from a tetraploid cell through a broad set of mechanisms [25]. Although chromosome lagging at anaphase was the most common type of cell division error found in NB cells in the present study, it does not exclude other mechanisms. Neither does our study provide evidence explaining the high frequency of anaphase lagging in NB cells. Multipolar cell divisions were found in some of the NB cell lines in the present study. It has been well demonstrated that multipolar cell divisions may re-orient to a pseudo-bipolar configuration before anaphase, which nevertheless shows a propensity for chromosome lagging due to merotelic spindle-kinetochore attachments [7], [8]. However, not all cell lines in the present study exhibited multipolar mitoses, while all of them still showed a high frequency of anaphase chromosome lagging. Centrosome disturbances, which are generally believed to be the main cause of multipolar mitoses, were evaluated in NBs by Fukushi et al. [35]. Although a connection could be established with ploidy divergence in a sub-group of infant NBs, they did not find the general connection to aneuploidy expected if extra centrosomes and spindle multipolarity was a major mechanism behind numerical aberrations in NB. Taken together, this makes centrosome disturbances a poor candidate behind the high frequency of lagging chromosomes in NB cells. However, we also found a high frequency of anaphase bridges, in accordance to a previous report showing critically short telomeres and chromosome fusion in NB [19]. Although this type of cell division anomaly has been intimately connected to structural chromosome aberrations [36], it has also been shown to result in loss of whole chromosomes [37], [38]. Another study [39] has also demonstrated a connection between telomere deficiency and tetraploidisation. This suggests that telomere dysfunction may contribute to aneuploidy in NB by causing chromosome loss and tetraploidisation in parallel. That telomere dysfunction is a common feature in many cancers has previously been shown by numerous studies [19], [40]–[43]. Whether telomere deficiency also has a role for the generation of aneuploidy in NB remains to be studied further.

In conclusion, we find that aneuploid NBs typically exhibit prominent intra-tumour genomic diversity with respect to both numerical and structural aberrations. For numerical aberrations, this diversity could be explained by loss of chromosomes at mitosis, in turn imposing a selection pressure towards polyploidisation, leading to even more complex scenarios of aneuploidy and clonal diversity.

## Supporting Information

Table S1 **Fluorescence in situ hybridization data from primary tumours and cell lines.** This table shows the proportion of cells with copy numbers different from the modal number (most common copy number) as well as the proportion deviating from the normal disomic state.
(DOCX)

Dataset S1 **SNP array data from 30 NBs used for estimation of tumour content by mBAF values.** Data Sheet 1 contains SNP array raw data output from the BAF segmentation algorithm. Column headings are annotated as follows: Chromosome = chromosomal location of each segment; Start = genome position of the first altered SNP marker according to GRCh37/hg19; End = genome position of last altered SNP marker; mBAF = segment median mirrored B-allele frequency; Log2ratio = median segment copy log2 copy number ratio. Data Sheet 2 contains curated segments with their estimated prevalence in each tumour sample. Column headings are annotated as follows: Case = case number in study; chromosome/segment, copy number aberrations annotated by cytogenetic nomenclature for segmental changes and indicated by CHR for whole chromosome alterations; mBAF, median mirrored B-allele frequency; mBAF min, minimum BAF within in segment; mBAF max, maximum BAF within segments, nB, number of B-alleles; nA, number of A-alleles; Mean AC (%), mean estimated tumour content of aberration; Min AC (%), minimum estimated tumour content of aberration; Max AC (%), maximum estimated tumour content of aberration; N segments, total number of abnormal segments; N numerical, total number of whole chromosome changes; N structural, total number of segmental chromosome changes; Span Total, difference in AC(%) between the most and least prevalent aberration; Span Numerical, difference in AC(%) between the most and least prevalent whole chromsosome aberration; Span Structural, difference in AC(%) between the most and least prevalent segmental aberration; Ploidy, given as multiples of the haploid state (n); Type, clinical-genetic subtype. Full SNP array profiles are not reported due to ethical issues arising from the risk of cross-study traceability.
(XLS)

Dataset S2 **Copy number profiles derived from reported neuroblastoma karyotypes.** The first column specifies the total chromosome number per case, the last specifies which clinical-genetic subtype it belongs to, and the centre columns give the distributions of chromosome copy numbers.
(XLS)

Dataset S3 **Copy number profiles derived from reported SNP array data.** The first column specifies the total chromosome number per case, the last specifies which clinical-genetic subtype it belongs to, and the centre columns give the distributions of chromosome copy numbers. Full SNP array profiles are not reported due to ethical issues arising from the risk of cross-study traceability.
(XLS)

Video S1 **Time lapse phase contrast microscopy of dividing GI-MEN cells.** See legend to Figure 1C for details.
(MOV)

Video S2 **Figure 9** **. Dynamic modelling of neuroblastoma genome evolution.** The video shows the evolution of chromosome numbers and predicted proliferative capacity for Pop1 and Pop2 over 500 generations of synchronized growth. See legend to Figure 9 for annotations and details.
(MOV)
