# Supplementary material for: Intratumour Diversity of Chromosome Copy Numbers in Neuroblastoma Mediated by On-Going Chromosome Loss from a Polyploid State
Source: PLoS One. 2013 Mar 21;8(3):e59268. doi: 10.1371/journal.pone.0059268 (PMC3605453; doi:10.1371/journal.pone.0059268)
Supplement: Table S1 — Fluorescence in situ hybridization data from primary tumours and cell lines. This table shows the proportion of cells with copy numbers different from the modal number (most common copy number) as well as the proportion deviating from the normal disomic state. (DOCX) [file pone.0059268.s001.docx]

**Table S1. Fluorescence in situ hybridization data from primary tumours and cell lines^1^**

| Genetic type |  | CEP 1 | CEP 2 | CEP 4 | CEP 7 | CEP 11 | CEP 12 | CEP 16 | CEP 17 | CEP 18 | 1p | 11q | 17q |
| --- | --- | --- | --- | --- | --- | --- | --- | --- | --- | --- | --- | --- | --- |
|  | **Adrenal gland** |  |  |  |  |  |  |  |  |  |  |  |  |
|  | n= | 2 | 2 | 2 | 2 | 2 | 2 | 2 | 2 | 2 | 2 | 2 | 2 |
|  | non-modal (%) | 3 | 1 | 0.5 | 3 | 0 | 0 | 2 | 2 | 3 | 0 | 0 | 0 |
|  | aneusomy (%) | 3 | 1 | 0.5 | 3 | 0 | 0 | 2 | 2 | 3 | 0 | 0 | 0 |
|  |  |  |  |  |  |  |  |  |  |  |  |  |  |
| 1 | **NRC 1** |  |  |  |  |  |  |  |  |  |  |  |  |
|  | n= | 4 | 5 |  |  | 4 | 4 | 3 |  | 3 |  |  |  |
|  | non-modal (%) | 59 | 56 |  |  | 47 | 66 | 49 |  | 47 |  |  |  |
|  | aneusomy (%) | 97 | 93 |  |  | 96 | 97 | 73 |  | 93 |  |  |  |
|  |  |  |  |  |  |  |  |  |  |  |  |  |  |
| 1 | **NRC 2^2^** |  |  |  |  |  |  |  |  |  |  |  |  |
|  | n= | 3 | 3 | 2 | 3 | 3 | 3 |  |  | 2 |  |  |  |
|  | non-modal (%) | 49 | 44 | 38 | 24 | 45 | 51 |  |  | 51 |  |  |  |
|  | aneusomy (%) | 58 | 67 | 38 | 82 | 71 | 56 |  |  | 49 |  |  |  |
|  |  |  |  |  |  |  |  |  |  |  |  |  |  |
| 1 | **NRC 3** |  |  |  |  |  |  |  |  |  |  |  |  |
|  | n= | 3 |  |  |  |  | 3 |  | 2 |  |  |  |  |
|  | non-modal (%) | 55 |  |  |  |  | 57 |  | 64 |  |  |  |  |
|  | aneusomy (%) | 75 |  |  |  |  | 79 |  | 64 |  |  |  |  |
|  |  |  |  |  |  |  |  |  |  |  |  |  |  |
| 1 | **NRC 5** |  |  |  |  |  |  |  |  |  |  |  |  |
|  | n= | 3 | 3 |  |  |  | 3 |  |  |  |  |  |  |
|  | non-modal (%) | 48 | 63 |  |  |  | 38 |  |  |  |  |  |  |
|  | aneusomy (%) | 79 | 88 |  |  |  | 76 |  |  |  |  |  |  |
|  |  |  |  |  |  |  |  |  |  |  |  |  |  |
| 2A | **NRC 7** |  |  |  |  |  |  |  |  |  |  |  |  |
|  | n= | 3 |  |  |  |  | 3 |  | 3 |  |  | 1 |  |
|  | non-modal (%) | 39 |  |  |  |  | 36 |  | 31 |  |  | 13 |  |
|  | aneusomy (%) | 82 |  |  |  |  | 77 |  | 70 |  |  | 90 |  |
|  |  |  |  |  |  |  |  |  |  |  |  |  |  |
| 2A | **NRC 13** |  |  |  |  |  |  |  |  |  |  |  |  |
|  | n= |  |  |  |  |  |  |  |  | 3 |  |  | 5 |
|  | non-modal (%) |  |  |  |  |  |  |  |  | 55 |  |  | 72 |
|  | aneusomy (%) |  |  |  |  |  |  |  |  | 78 |  |  | 94 |
|  |  |  |  |  |  |  |  |  |  |  |  |  |  |
| 2A | **SK-N-AS** |  |  |  |  |  |  |  |  |  |  |  |  |
|  | n= |  |  |  | 2 | 2 |  |  | 1 | 2 | 2 | 1 | 4 |
|  | non-modal (%) |  |  |  | 34 | 14 |  |  | 26 | 32 | 16 | 18 | 73 |
|  | aneusomy (%) |  |  |  | 34 | 14 |  |  | 76 | 32 | 16 | 82 | 93 |
|  |  |  |  |  |  |  |  |  |  |  |  |  |  |

**Table S1 - *continued***

| Genetic type |  | CEP 1 | CEP 2 | CEP 4 | CEP 7 | CEP 11 | CEP 12 | CEP 16 | CEP 17 | CEP 18 | 1p | 11q | 17q |
| --- | --- | --- | --- | --- | --- | --- | --- | --- | --- | --- | --- | --- | --- |
| 2A | **SK-N-SH** |  |  |  |  |  |  |  |  |  |  |  |  |
|  | n= |  |  |  | 3 | 2 |  |  | 2 | 2 | 2 |  | 4 |
|  | non-modal (%) |  |  |  | 27 | 10 |  |  | 10 | 25 | 8 |  | 4 |
|  | aneusomy (%) |  |  |  | 88 | 10 |  |  | 10 | 25 | 8 |  | 79 |
|  |  |  |  |  |  |  |  |  |  |  |  |  |  |
| 2B | **NRC 4** |  |  |  |  |  |  |  |  |  |  |  |  |
|  | n= | 2 |  |  |  |  | 2 |  |  | 2 | 1 |  | 3 |
|  | non-modal (%) | 3 |  |  |  |  | 4 |  |  | 11 | 6 |  | 50 |
|  | aneusomy (%) | 3 |  |  |  |  | 4 |  |  | 11 | 94 |  | 94 |
|  |  |  |  |  |  |  |  |  |  |  |  |  |  |
| 2B | **NRC 8** |  |  |  |  |  |  |  |  |  |  |  |  |
|  | n= |  |  |  |  | 4 |  | 3 |  |  |  |  | 5 |
|  | non-modal (%) |  |  |  |  | 66 |  | 57 |  |  |  |  | 75 |
|  | aneusomy (%) |  |  |  |  | 96 |  | 57 |  |  |  |  | 84 |
|  |  |  |  |  |  |  |  |  |  |  |  |  |  |
| 2B | **IMR32** |  |  |  |  |  |  |  |  |  |  |  |  |
|  | n= |  |  |  | 2 | 2 | 3 |  | 3 | 2 | 2 |  | 4 |
|  | non-modal (%) |  |  |  | 20 | 36 | 28 |  | 33 | 4 | 32 |  | 48 |
|  | aneusomy (%) |  |  |  | 20 | 36 | 80 |  | 72 | 4 | 32 |  | 80 |
|  |  |  |  |  |  |  |  |  |  |  |  |  |  |
| 2B | **GI-M-EN** |  |  |  |  |  |  |  |  |  |  |  |  |
|  | n= |  |  |  |  | 2 |  |  | 2 | 3 | 2 |  | 2 |
|  | non-modal (%) |  |  |  |  | 51 |  |  | 36 | 23 | 8 |  | 48 |
|  | aneusomy (%) |  |  |  |  | 51 |  |  | 36 | 80 | 8 |  | 48 |
|  |  |  |  |  |  |  |  |  |  |  |  |  |  |
| 2B | **SK-N-FI** |  |  |  |  |  |  |  |  |  |  |  |  |
|  | n= |  |  |  | 2 | 2 |  |  |  | 2 | 2 |  | 3 |
|  | non-modal (%) |  |  |  | 4 | 15 |  |  |  | 18 | 9 |  | 58 |
|  | aneusomy (%) |  |  |  | 4 | 15 |  |  |  | 18 | 9 |  | 58 |

^1^ Columns correspond to results for centromere probes (CEP) and probes for chromosome segments 1p, 11q, and 17q. The first row under each specimen denotes the modal number of each particular chromosome or segment, second row is the percentage of nuclei not having the modal copy number and the final row the percentage of nuclei having aneusomi (e.g. non-disomy). Results above the mean aneusomy for all probes in normal adrenal tissue +3 standard deviations (6.5%) are denoted in red, corresponding to a significant prevalence of cells with non-modal chromosome number. ^2^ SNP array data not available due to difficulties in interpretation of modal values.
